# Identification of Chloride Intracellular Channel Protein 3 as a Novel Gene Affecting Human Bone Formation

**DOI:** 10.1002/jbm4.10003

**Published:** 2017-04-28

**Authors:** Andrea M Brum, Cindy S van der Leije, Marijke Schreuders‐Koedam, Jeroen Verhoeven, Mark Janssen, Dick HW Dekkers, Jeroen AA Demmers, Marco Eijken, Jeroen van de Peppel, Johannes PTM van Leeuwen, Bram CJ van der Eerden

**Affiliations:** ^1^ Department of Internal Medicine School of Molecular Medicine Erasmus University Medical Center Rotterdam the Netherlands; ^2^ Arcarios BV Rotterdam the Netherlands; ^3^ Proteomics Center Erasmus University Medical Center Rotterdam The Netherlands

**Keywords:** CLIC3, OSTEOBLAST, MESENCHYMAL STEM CELL, DIFFERENTIATION, BONE, LINEAGE DECISION MAKING

## Abstract

Osteoporosis is a common skeletal disorder characterized by low bone mass leading to increased bone fragility and fracture susceptibility. The bone building cells, osteoblasts, are derived from mesenchymal stromal cells (MSCs); however, with increasing age osteogenic differentiation is diminished and more adipocytes are seen in the bone marrow, suggesting a shift in MSC lineage commitment. Identification of specific factors that stimulate osteoblast differentiation from human MSCs may deliver therapeutic targets to treat osteoporosis. The aim of this study was to identify novel genes involved in osteoblast differentiation of human bone marrow–derived MSCs (hMSCs). We identified the gene chloride intracellular channel protein 3 (*CLIC3*) to be strongly upregulated during MSC‐derived osteoblast differentiation. Lentiviral overexpression of *CLIC3* in hMSCs caused a 60% increase of matrix mineralization. Conversely, knockdown of *CLIC3* in hMSCs using two short‐hairpin RNAs (shRNAs) against *CLIC3* resulted in a 69% to 76% reduction in *CLIC3* mRNA expression, 53% to 37% less alkaline phosphatase (ALP) activity, and 78% to 88% less matrix mineralization compared to scrambled control. Next, we used an in vivo human bone formation model in which hMSCs lentivirally transduced with the *CLIC3* overexpression construct were loaded onto a scaffold (hydroxyapatite‐tricalcium‐phosphate), implanted under the skin of NOD‐SCID mice, and analyzed for bone formation 8 weeks later. *CLIC3* overexpression led to a 15‐fold increase in bone formation (0.33% versus 5.05% bone area relative to scaffold). Using a Clic3‐His‐tagged pull‐down assay and liquid chromatography–mass spectrometry (LS/MS)‐based proteomics analysis in lysates of osteogenically differentiated hMSCs, we showed that CLIC3 interacts with NIMA‐related kinase 9 (NEK9) and phosphatidylserine synthase 1 (PTDSS1) in vitro, and this finding was supported by immunofluorescent analysis. In addition, inhibition of *NEK9* or *PTDSS1* gene expression by shRNAs inhibited osteoblast differentiation and mineralization. In conclusion, we successfully identified CLIC3 to be a lineage‐specific gene regulating osteoblast differentiation and bone formation through its interaction with NEK9 and PTDSS1. © The Authors. *JBMR Plus* is published by Wiley Periodicals, Inc. on behalf of the American Society for Bone and Mineral Research.

## Introduction

Bone is a dynamic organ that throughout life undergoes constant remodeling through bone formation by osteoblasts and bone resorption by osteoclasts. Osteoporosis is a common skeletal disease characterized by reduced bone mass and increased fragility and fracture risk, occurring when bone remodeling is disrupted and bone resorption overtakes bone formation.^1^ The vast majority of osteoporosis treatments, such as bisphosphonates, act through reduction of bone resorption, and result in modest increases in bone density; however, these treatments do not result in a true bone anabolic effect so patients do not regain the bone that has been lost at time of diagnosis. An ideal treatment would also stimulate osteoblast differentiation and/or bone formation to help repair the already damaged bone microarchitecture. Identification of novel genes or processes that stimulate osteoblast differentiation may therefore deliver therapeutic targets for the development of novel treatments for bone diseases such as osteoporosis.

Osteoblasts are derived from the bone marrow mesenchymal stem, or stromal, cells (MSCs) and undergo terminal differentiation to form osteocytes.^2^ Besides osteoblasts, MSCs give rise to other cell types, including adipocytes and chondrocytes.^3^ Osteogenic differentiation of MSCs is a tightly regulated process that progresses through several phases. MSCs are driven to the osteogenic lineage by expression of the osteoblast‐specific transcription factors Runx2 and Osterix.^4^, ^5^, ^6^ After initial commitment, differentiation toward mature osteoblasts is typically characterized by the expression of collagen type I, osteocalcin, osteopontin, bone sialoprotein, and alkaline phosphatase (ALP).^7^ However, the precise mechanism of how human bone marrow–derived MSCs (hMSCs) differentiate into osteoblasts is still largely unknown, including the exact mechanism by which glucocorticoids stimulate osteoblast differentiation in vitro.^8^, ^9^

With aging there is a decrease in bone mass and increased bone fragility, while at the same time there is an increase in total and bone marrow adiposity.^10^, ^11^ As both the osteogenic and adipogenic lineages derive from the same progenitor, it appears that there is a shift in the lineage decision‐making process of MSCs with increasing age. This theory is supported by previous work showing that bone marrow–derived mesenchymal progenitors from aged humans, rats, and mice preferentially differentiate toward the adipogenic, rather than osteogenic, lineage.^10^, ^11^, ^12^, ^13^, ^14^

Our aim was to expand the knowledge of human osteoblastogenesis by identifying novel genes and processes involved in differentiation of hMSCs to the osteogenic lineage. Gene expression analysis of hMSCs undergoing osteogenic and adipogenic differentiation led us to study chloride intracellular channel protein 3 (*CLIC3*) as an osteogenic lineage‐specific candidate. CLIC proteins are a conserved family of proteins first identified when a novel chloride ion channel was discovered in bovine kidney.^15^, ^16^ This family of proteins can transition between a soluble globular form and an integral membrane protein that is able to mediate ion conductance and/or ion channel formation.^17^, ^18^ However, their role as channel proteins under physiological conditions has been strongly debated. To date, six CLIC paralogues have been identified in vertebrates, all of which appear to have diverse functions.^18^, ^19^ CLIC3 is a largely unstudied 26.6‐kDa member of the CLIC family. It has been implicated to be involved in cellular processes including integrin recycling,^20^, ^21^ endosome trafficking,^22^ and cell growth via interactions with ERK7.^23^

## Materials and Methods

### Cell culture

hMSCs were cultured as described^24^ and are detailed in the Supporting Information.

### Lentiviral‐mediated overexpression and knockdown

To obtain *CLIC3* overexpression, we generated full‐length human *CLIC3* cDNA (Open Biosystems; GE Dharmacon, Lafayette, CO) containing a His‐tag stop codon into a pEntr vector and verified using proofreading PCR (Table 1). hMSCs cells were transduced with the *CLIC3* vector or empty vector (pLenti6.3 vector without CLIC3 construct), as control. For gene knockdown of *CLIC3*, NIMA‐related kinase 9 (*NEK9*), and phosphatidylserine synthase 1 (*PTDSS1*), constructs from the TRC‐Hs1.0 library (Sigma‐Aldrich, Zwijndrecht, the Netherlands) were used (Table 2). A nontargeting short‐hairpin RNA (shRNA) vector with a scrambled sequence (Table 2) was used as a negative control. Methods for gene overexpression and silencing have been described^25^, ^26^ and are specified in the Supporting Information.

**Table 1:** Oligonucleotide Primers Used for Proofreading PCR for Generation of His‐Tagged CLIC3 Overexpression Vector

Gene	Forward (5′–3′)	Reverse (5′–3′)
*CLIC3‐His*	CACCATGGCGGAGACCAAGCTCCA	TCATTACTAGTGATGGTGATGGTGATGGCGGGGTGCACGGCGGGCC

Sequences of forward and reverse primers used for proofreading PCR in this study. Detected using SYBR green.

**Table 2:** List of shRNAs Used for *CLIC3*, *NEK9*, and *PTDSS1*

Target gene	ID	Target sequence
*CLIC3*	sh9123	GCCTCGTTACAGGGAGTCCAA
	sh9126	GCAGGAGAAAGAGTTCAAATA
*NEK9*	sh10	GCCTTGATTATTGTTGCAGTT
	sh11	CCGAGGAATGGAAGGTTTAAT
	sh12	CCAAAGGAACTCAGACAGCAA
	sh13	GTGAAGATCGTGCAAGGAATT
	sh14	GTACATTTGGAGAGTGGCATT
*PTDSS1*	sh25	CGAGCAGGTTAAATCTCTAAT
	sh26	GCTAGATCCAAATCTTCGATA
	sh27	TGGACCTATGTTCGATGGTTT
	sh28	GCAACAACGAAAGCCATTCTT
	sh29	GACTGAGTTGAATACCTTCTT
Nontargeting shRNA	ID	Oligonucleotide sequence
Scrambled	SHC002	CCGGCAACAAGATGAAGAGCACCAACTCGAGTTGGTGCTCTTCATCTTGTTGTTTTT

Target sequences of shRNAs for gene silencing of *CLIC3*, *NEK9*, and *PTDSS1*, as well as the oligonucleotide sequence for a nontargeting scrambled control.

### ALP, mineralization, and protein assays

ALP and calcium measurements were performed as described^8^, ^24^ and are described in detail in the Supporting Information.

### Western blotting

Immunodetection of CLIC3 by Western blot was performed as described^27^ and is further detailed in the Supporting Information. The membrane was incubated with a specific antibody against CLIC3 (ab56364; Abcam, Cambridge, UK; 1:500). Membranes were probed with the secondary antibody goat anti‐mouse conjugated with Alexa Fluor 680 (1:5000; Invitrogen/Fisher Scientific, Landsmeer, Netherlands; Cat. A21057).

### Quantification of mRNA expression

RNA isolation, cDNA synthesis, and PCR reactions were performed as described.^24^ Oligonucleotide primer pairs were designed to be either on exon boundaries or spanning at least one intron (Table 3). Gene expressions were corrected for expression of the housekeeping gene *GAPDH*. Experiments were performed at least in duplicate in a minimum of two separate experiments.

**Table 3:** Oligonucleotide Primers Used in the Study

Gene	Forward (5′–3′)	Reverse (5′–3′)
*GAPDH*	CCGCATCTTCTTTTGCGTCG	CCCAATACGACCAAATCGTTG
*CLIC3*	CTGCCCATCCTGCTCTAT	CAGCGTCTCCTCCAGAAA
*NEK9*	TCAGCAATCCAGTGGAGCAG	CCAGTCGTCCATATTCGCCA
*PTDSS1*	TCGCCTTTACCAGGGATGAC	GAGTGAACGGACCATTGGGG

Sequences of forward and reverse primers used for qPCR in this study. All genes were detected using SYBR green.

### Immunocytochemistry

Detection of CLIC3, PTDSS1, and NEK9 by immunocytochemistry was performed as described^28^; specific information can be found in the Supporting Information. Primary antibodies used were: anti‐CLIC3 raised in mouse, 1:150 (ab56364; Abcam, Cambridge, UK); anti‐NEK9 raised in rabbit, 1:20 (11192‐1‐AP; Proteintech, Manchester, UK); anti‐PTDSS1 raised in rabbit, 1:50 (HPA016852; Atlas Antibodies Bromma, Sweden).

### In vivo implantation assay

All animal experiments were performed in compliance with the animal ethics board of the Erasmus Medical Center. Experiments were performed based on the protocol by Abdallah and colleagues^29^ and details can be found in the Supporting Information. Five healthy, adult, female NOD.CB17‐Prkdc^scid^/NCrHsd (NOD‐SCID) mice (Charles River Laboratories, 's‐ Hertogenbosch, Netherlands) were used for these studies; each mouse had up to four implants surgically inserted subcutaneously under anesthesia. Quantification was carried out using Image J software (NIH, Bethesda, MD, USA; https://imagej.nih.gov/ij/): ceramics areas and bone areas were determined by eye based on staining and morphology, the edges were hand drawn and the resulting pixel measurements were calculated back to mm^2^. The observer assessing the pellets was blinded toward their identity.

### Pull‐down assay

A pull‐down assay was performed to isolate proteins associating with His‐tagged CLIC3 using Dynabeads (10103D; Life Technologies, Bleiswijk, Netherlands). Purification of CLIC3 protein complexes was performed by a metal‐based affinity between the Dynabeads and the His‐tagged CLIC3. The pull‐down was performed according to the manufacturers’ protocol and exact details can be found in the Supporting Information. Bait protein was obtained from hMSCs differentiated with dexamethasone for 5 days, following transduction with either His‐tagged *CLIC3* or an empty vector (EV; control). Two replicate samples from each condition were included for subsequent mass spectrometry (MS) assessment.

### MS

MS detection of proteins binding to CLIC3‐His was performed as described^30^ and is described in full in the Supporting Information.

### Bioinformatic analysis

The raw MS data were analyzed by MaxQuant software (version 1.3.0.5).^31^ A false‐discovery rate of 0.01 for proteins and peptides and a minimum peptide length of six amino acids were required. The Andromeda search engine^32^ was used to search the MS/MS spectra against the Uniprot database (taxonomy: *Homo sapiens*, release HUMAN_2013_04) concatenated with the reversed versions of all sequences (maximum of two missed cleavages; 0.6‐Da fragment mass tolerance, enzyme specificity: trypsin). The data from the replicates were combined as averages. For selection of the most relevant interacting proteins the following criteria were set: (1) a label‐free quantification (LFQ) value in CLIC3 samples of greater than 1 × 10^6^; (2) number of unique peptides covering a protein equals three or more; and (3) ratio of CLIC3 samples versus control samples of 1.5 or greater. Proteins were then ranked based on the average LFQ ratio of the CLIC3 samples versus the control samples. Ingenuity Pathway Analysis (IPA) (http://www.ingenuity.com) and Gene Ontology analysis (GO) in DAVID (https://david.ncifcrf.gov) was performed using the 52 proteins identified by pull‐down and MS.

### Statistics

The data provided here are based on at least two independent experiments performed at least in duplicate. Values displayed are mean ± SE. Significance was calculated using either the Student's *t* test, Mann Whitney test, or the one‐way ANOVA with Tukey's post hoc test where appropriate, using GraphPad Prism 6.0 (GraphPad Software, Inc., La Jolla, CA, USA). Values of *p* <0.05 were considered significant.

## Results

### *CLIC3* is upregulated during human osteogenic differentiation and downregulated during adipogenesis

**Figure 1:**
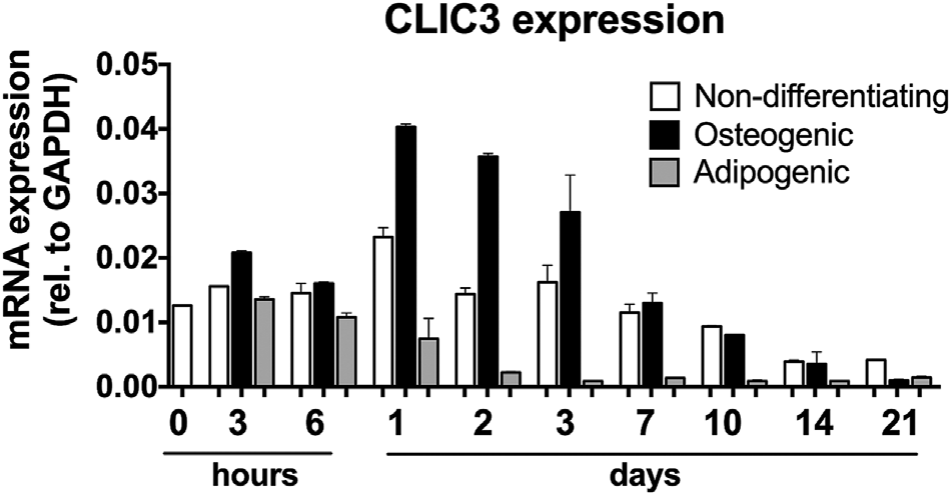
*CLIC3* expression is upregulated during osteoblast differentiation and downregulated during adipocyte differentiation. mRNA expression levels of *CLIC3* in hMSCs cultured in nondifferentiating (white bars), osteogenic (black bars), and adipogenic (gray bars) conditions over 3 weeks assessed by quantitative PCR. Graph displays a representative experiment. *n* = 2.

*CLIC3* is dynamically expressed in human mesenchymal stromal cells (hMSCs) during osteogenic differentiation (Fig. 1). Figure 1 shows that expression of *CLIC3* is increased during the osteogenic differentiation of hMSCs, peaking between 1 and 3 days of culture, compared to nondifferentiating. In addition we saw that *CLIC3* expression goes down in adipogenic cells compared to nondifferentiating, showing an opposite effect to that of osteoblasts. These data led us to further scrutinize *CLIC3* as a candidate gene for human osteoblastogenesis.

### Overexpression of *CLIC3* in hMSCs enhances in vitro osteogenic differentiation

**Figure 2:**
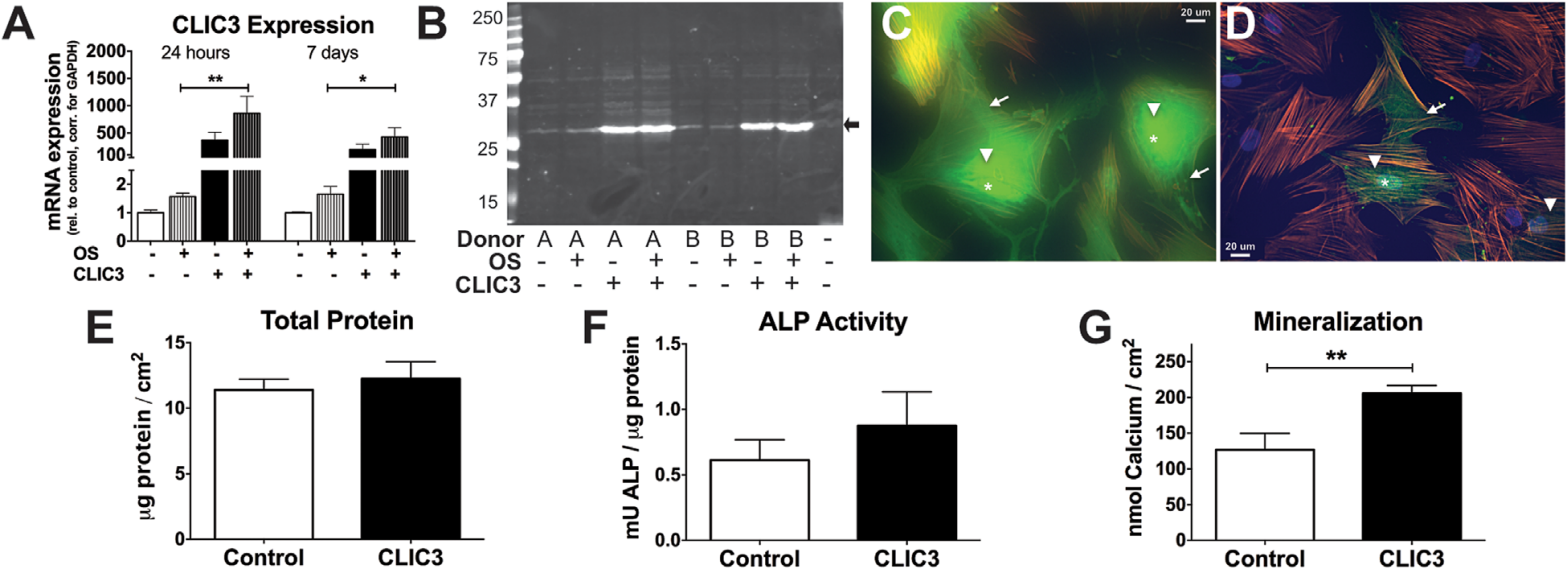
*CLIC3* overexpression enhances human osteoblast differentiation. Enhancement of *CLIC3* expression was assessed by (*A*) quantification of *CLIC3* mRNA expression by quantitative PCR of hMSCs transduced with empty vector (white bars) or *CLIC3* overexpression vector (black bars) under nondifferentiating (solid bars) or osteogenic differentiation (patterned bars) conditions at 24 hours (left 4 bars) or 7 days (right 4 bars) after the start of differentiation, or by (*B*) immunoblotting in protein lysates collected 10 days postinduction of differentiation in two hMSC donors (A and B) using anti‐CLIC3 antibody. Arrow indicates the predicted size of CLIC3 protein at 27 kDa, last lane contains loading buffer only. In additional CLIC3 expression was visualized by immunofluorescence microscopy using CLIC3 antibody in hMSCs differentiated to osteoblasts and transduced with *CLIC3* (*C*) or empty vector (*D*) at total magnification of ×400 after 4 days of differentiation. CLIC3, in green, is seen in the cytoplasm (arrows), the perinuclear region (arrowheads), and some nuclear staining (asterisk); actin cytoskeleton was visualized by phalloidin antibody in orange, and nuclei by DAPI in blue. Biochemical assays are shown for total protein (day 6) (*E*), ALP activity (day 6) (*F*), and mineralization (week 3) (*G*) of hMSCs transduced with *CLIC3* (black bars) or empty vector (white bars) and osteogenic culture conditions. Graphs display combined results of all experiments. (*A*) *n* = 8–12; (*E*,*F*) *n* = 18; (*G*) *n* = 19. *p < 0.05, **p < 0.01. OS = osteogenic media. CLIC3 = lentiviral transduction with *CLIC3* expression vector 1 day prior to the start of differentiation; DAPI = 4,6‐diamidino‐2‐phenylindole.

To determine the role CLIC3 has in human osteoblastogenesis we overexpressed *CLIC3* in hMSCs by lentiviral transduction and studied its effect on classical biochemical markers of osteoblast differentiation. Efficient overexpression of *CLIC3* in hMSCs was determined by gene expression analysis and immunoblotting. Figure 2 *A* demonstrates successfully elevated *CLIC3* mRNA expression by greater than 100‐fold at both day 1 and 7 of culture in both nondifferentiating and differentiating hMSCs. Concordantly, protein levels of CLIC3 were also strongly increased at day 10 of culture in nondifferentiating and differentiating hMSCs (Fig. 2 *B*). Immunofluorescent CLIC3 detection in differentiating hMSCs reveals that *CLIC3* overexpression (Fig. 2 *C*) intensifies the level of CLIC3 protein in most cells, compared to EV‐transduced cells (Fig. 2 *D*). CLIC3 expression is predominately localized to the perinuclear region (arrowheads; Fig. 2 *C*, *D*), In *CLIC3‐*overexpressing cells (Fig. 2 *C*) there is increased expression in the cytoplasm (arrows) and nucleus (asterisk) compared to control cells (Fig. 2 *D*). *CLIC3* overexpression did not affect total protein levels (Fig. 2 *E*) or ALP activity (Fig. 2 *F*) in osteogenic hMSCs, but it did enhance mineralization by 60% as shown by total calcium quantification after 3 weeks of culture (Fig. 2 *G*). Overall, these results show that we have successfully overexpressed *CLIC3* in hMSCs and that CLIC3 enhances in vitro mineralization.

### CLIC3 is critical for in vitro human osteogenic differentiation

**Figure 3:**
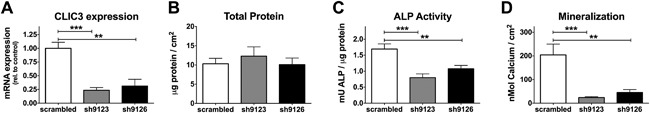
Knockdown of *CLIC3* expression inhibits human osteoblast differentiation. Transduction of hMSCs with 2 shRNAs against CLIC3 (shaded bars) reduces (*A*) mRNA expression of CLIC3 as determined by quantitative PCR (day 4), (*B*) total protein (day 6), (*C*) ALP activity (day 6), and (*D*) mineralization (week 3) compared to scrambled control (white bar). Graphs display combined results from all experiments. (*A*) *n* = 4–5; (*B*) *n* = 7; (*C*) *n* = 7; (*D*) *n* = 6. **p < 0.01, ***p < 0.001.

To determine if CLIC3 is essential for human osteoblast differentiation we used shRNAs against endogenous *CLIC3* to knockdown its expression in hMSCs. Two separate shRNAs decreased *CLIC3* expression in hMSCs by 76% and 69% (Fig. 3 *A*), respectively, compared to cells transduced with scrambled shRNA. Total protein levels were not affected by either shRNA (Fig. 3 *B*). Knockdown of *CLIC3* mRNA expression reduced ALP activity by 53% and 37%, respectively, after 1 week of culture (Fig. 3 *C*). After 3 weeks of culture in osteogenic medium, CLIC3 silencing reduced mineralization by approximately 80% compared to the scrambled control (Fig. 3 *D*).

### *CLIC3* enhances in vivo human bone formation

**Figure 4:**
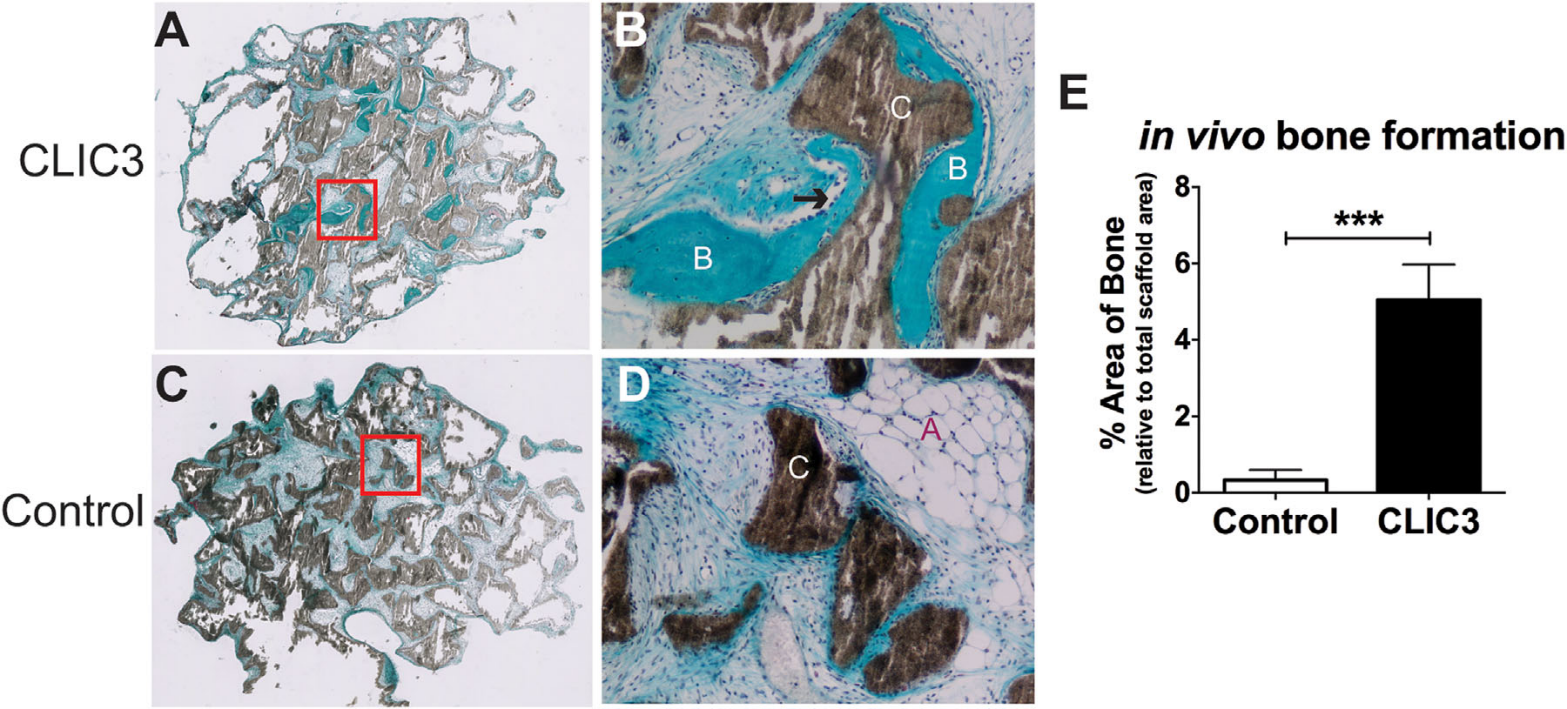
*CLIC3* promotes human bone formation in vivo. Histological sections of explants of *CLIC3* overexpressing hMSCs (*A*, low magnification; *B*, high magnification) implanted under the skin of immune deficient (NOD‐SCID) mice show more prevalent areas of new bone formation (labeled “B”) and osteoblasts (arrow) compared to empty vector‐transduced cells (*C*, low magnification; *D*, high magnification). HA‐TCP ceramic scaffold is labeled “C,” adipocytes are labeled “A.” (*E*) *CLIC3* overexpression in hMSCs (black bar) increases the amount of newly formed bone in explants compared to control cells (white bar) based on quantification of the area of new bone formed as a percentage of total scaffold area, using ImageJ software. *n* = 9–10. ***p < 0.001.

In order to test the in vivo effect of *CLIC3* manipulation on human bone formation we subcutaneously implanted hMSCs transduced with *CLIC3* or empty vector (control) loaded onto a hydroxyapatite/tricalcium phosphate (HA/TCP) scaffold in immune‐deficient mice and quantified the amount of heterotopic bone formed. As shown in Fig. 4, hMSCs overexpressing *CLIC3* formed a significantly greater amount of ectopic bone compared to MSCs transduced with an empty vector. This is visualized in the representative Goldner‐stained sections made from implants containing *CLIC3* overexpressing hMSCs (Fig. 4 *A*, *B*) containing patches of newly formed bone and osteoblasts compared to the control‐treated hMSC‐implants (Fig. 4 *C*, *D*) showing low level of newly formed bone. Quantification of the amount of new bone formed revealed that implants from *CLIC3*‐overexpressing hMSCs contain 15 times more bone compared to implants containing control‐treated cells (Fig. 4 *E*). These results clearly show that *CLIC3* enhances in vivo bone human formation.

### CLIC3 pull‐down identifies 52 proteins as potential interacting with CLIC3 in differentiating osteoblasts

In an attempt to gain insight into the molecular interactions by which CLIC3 acts on osteoblasts, we performed a pull‐down assay followed by MS analysis to identify which proteins in cell extracts of hMSCs differentiated toward osteoblasts associate with CLIC3. Supporting Table  1 lists all CLIC3‐His pulled‐down proteins ranked according to the average LFQ intensity ratio of CLIC3‐His–baited samples versus control samples, which are based on nonspecific binding. Bioinformatic analyses of these proteins were performed using Ingenuity Pathway Analysis and showed that “PRPP biosynthesis I,” “gap junction signaling,” “paxillin signaling,” “integrin signaling,” and various endocytosis and exocytosis pathways comprise the most significant canonical pathways in the list of proteins (summarized in Table 4, detailed in Supporting Table 2). Using DAVID Gene Ontology analysis, the most significant GO terms included processes related to RNA nuclear export and localization, transport, cell adhesion, and localization to the nuclear periphery and pores (summarized in Table 4, detailed in Supporting Table 3). Out of the 52 proteins identified by CLIC3‐His pull‐down, eight proteins were uniquely present in our CLIC3 samples, as compared to control samples (Table 5), and we chose to analyze two of these proteins further: NEK9 and PTDSS1.

**Table 4:** Summary of Top of Canonical Pathways and GO Terms

Canonical pathway^a^	Biological process^b^	Molecular function^b^	Cellular component^b^
PRPP biosynthesis	Export from nucleus	RNA binding	Adherens cell junction
Gap junction signaling	RNA localization	Cadherin binding	Cytosol
Paxillin signaling	Transport	Cell adhesion: protein and molecule binding	Nuclear periphery/pore
Integrin signaling	Adhesion		
Endocytosis/exocytosis			

Top canonical pathways determined by Ingenuity pathway analysis from the 52 proteins found to pull down with Clic3‐His. GO term clusters that have been identified by DAVID bioinformatics resources for the same proteins. The GO terms within the clusters had a significance of Benjamini corrected *p* value <0.05. The headers “biological process,” “molecular function,” and “cellular components” stand for the GO category to which the GO term is annotated.

^a^ Determined by Ingenuity pathway analysis (http://www.ingenuity.com/).

^b^ Determined by DAVID Functional Annotation Analysis (http://david.ncifcrf.gov).

**Table 5:** Top Proteins Identified in CLIC3‐His Pull‐Down

Protein	Gene symbol	LFQ CLIC3_A	LFQ CLIC3_B	LFQ EV_A	LFQ EV_B	Number of unique peptides	Ratio LFQ CLIC3:EV
Chloride intracellular channel protein 3	CLIC3	1.27E+09	1.19E+09	nd	nd	15	1.23E+09
Serine/threonine‐protein kinase Nek9	NEK9	6.31E+06	5.12E+06	nd	nd	3	5.71E+06
1‐Phosphatidylinositol 4,5‐bisphosphate phosphodiesterase beta‐3	PLCB3	2.84E+06	4.29E+06	nd	nd	7	3.56E+06
Aminoacyl tRNA synthase complex‐interacting multifunctional protein 1	AIMP1	2.49E+06	3.29E+06	nd	nd	3	2.89E+06
Casein kinase II subunit alpha 3; alpha 1	CSNK2A1; CSNK2A3	1.70E+06	3.00E+06	nd	nd	3	2.35E+06
40S Ribosomal protein S28	RPS28	1.64E+06	1.97E+06	nd	nd	3	1.80E+06
Phosphatidylserine synthase 1	PTDSS1	1.21E+06	1.73E+06	nd	nd	3	1.47E+06
Nuclear pore complex protein Nup160	NUP160	1.15E+06	1.06E+06	nd	nd	3	1.11E+06

Proteins identified by MS from CLIC3‐His pull‐down assay in hMSCs undergoing osteogenic differentiation. Protein lysates from osteogenically differentiated hMSCs were isolated on day 5 in cultures either overexpressing His‐tagged *CLIC3* or transduced with EV control, and subjected to protein pull‐down for the His‐tagged CLIC3 and proteins binding to it. Proteins were determined by mass spectrometry measurements (*n* = 2). The top proteins are listed here, ranked on their ratio of CLIC3 versus control average LFQ intensity (ie, present in CLIC3 overexpressing condition and absent in the EV condition).

LFQ = label‐free quantification; EV = empty vector; nd = not detected.

### CLIC3 interacts with NEK9 and PTDSS1 during osteogenic differentiation of MSCs

**Figure 5:**
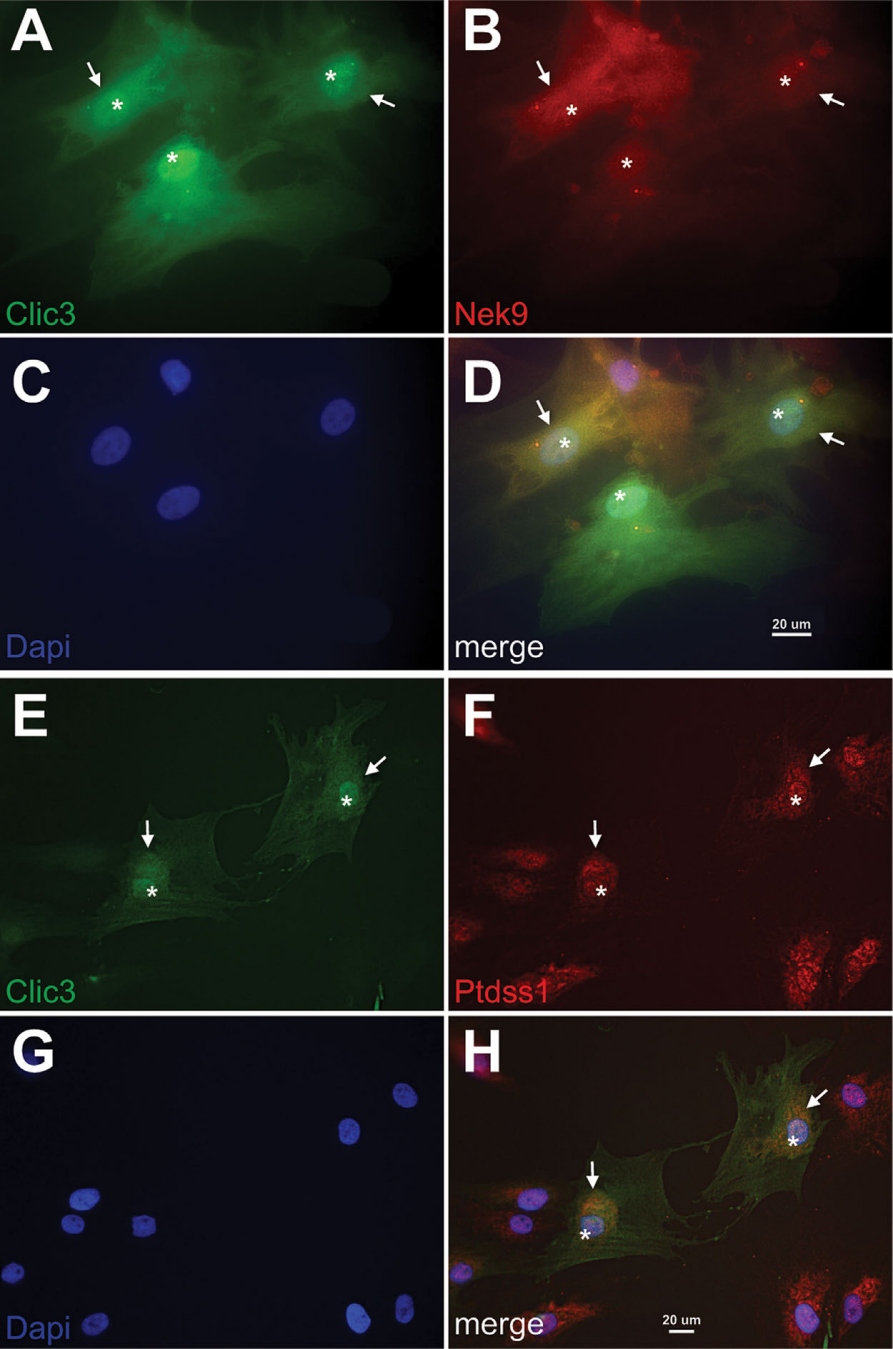
CLIC3 colocalizes with PTDSS1 and NEK9 in differentiating osteoblasts. Using immunofluorescence microscopy, CLIC3 (*A*, *D*, green) and NEK9 (*B*, *D*, red) were visualized together in hMSCs differentiated toward osteoblast and overexpressing CLIC3 at magnification ×630. *C*, *D* show nuclear staining by DAPI in blue. Arrows indicate detection of both proteins in the perinuclear region and asterisks denote nuclear localization. Immunofluorescent detection of CLIC3 (*E*, *H*, green) and PTDSS1 (*F*, *H*, red) were visualized jointly in osteogenic hMSCs overexpressing CLIC3 at magnification ×400. *G*, *H* show nuclear staining by DAPI in blue. PTDSS1 and CLIC3 are both seen in the perinuclear (arrows) and nuclear (asterisks) compartments. DAPI = 4,6‐diamidino‐2‐phenylindole.

To confirm the colocalization of CLIC3 with the proteins identified by the pull‐down assay we performed immunofluorescent analysis. At day 5 of differentiation, CLIC3 (Fig. 5 *A*) and NEK9 (Fig. 5 *B*) colocalize in the perinuclear region (arrows, Fig. 5 *D*) in *CLIC3* overexpressing hMSCs, with some CLIC3 expression seen in the nucleus as well (asterisk, Fig. 5 *A*), overlapping with DAPI nuclear staining (5C,D).

Similar to Fig. 5 *A*, Fig. 5 *E* exhibits the cytosolic (arrowhead), perinuclear (arrows), and nuclear distribution of CLIC3 in osteogenically differentiated hMSCs, whereas PTDSS1 is localized specifically to the perinuclear (arrows) and nuclear (asterisk, overlapping with DAPI nuclear staining (5G)) compartments (Fig. 5 *F*), and colocalization is seen in the perinuclear region (Fig. 5 *H*).

### Inhibition of *NEK9* and *PTDSS1* affects osteogenic differentiation of hMSCs

**Figure 6:**
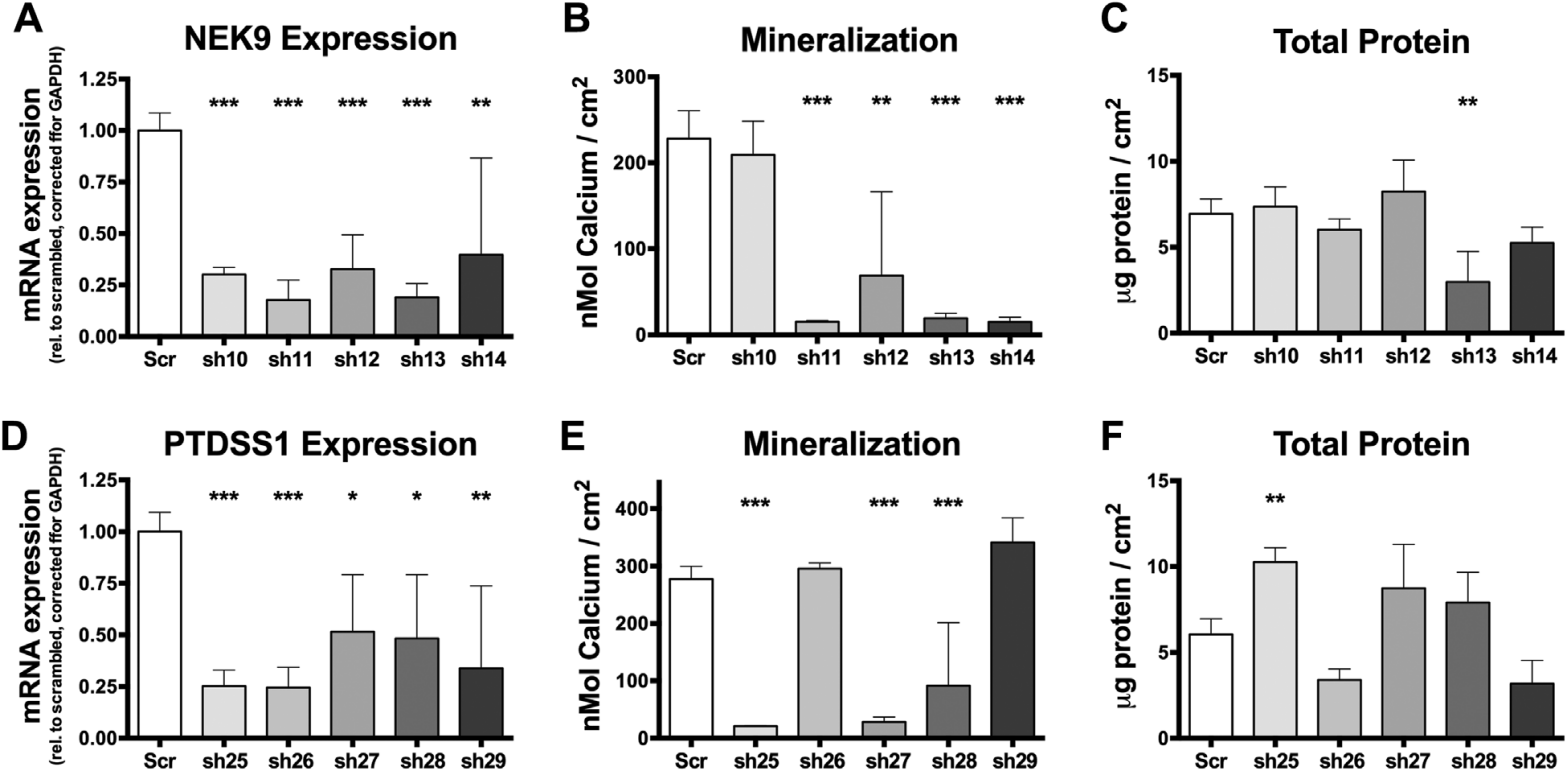
Knockdown of *NEK9* and *PTDSS1* gene expression inhibits osteogenic differentiation of hMSCs. mRNA expression of *NEK9* (*A*) as determined by quantitative PCR (day 3), mineralization (*B*) (week 3), and total protein quantification (*C*) (week 3) after transduction of hMSCs with five shRNAs against *NEK9* (shaded bars) compared to scrambled control (white bars). mRNA expression of *PTDSS1* (*D*) as determined by quantitative PCR (day 3), mineralization (*E*) (week 3), and total protein quantification (*F*) (week 3) after transduction of hMSCs with five shRNAs against *PTDSS1* (shaded bars) compared to scrambled control (white bars). Graphs display combined results from all experiments. (*A*) *n* =  4–12; (*B*) *n* = 6–12; (*C*) *n* = 6–12; (*D*) *n* = 4–12; (*E*) *n* = 5–12; (*C*) *n* = 6–12. *p < 0.05, **p < 0.01, ***p < 0.001.

To determine if NEK9 or PTDSS1 play a role in human osteoblast differentiation, we studied the effects of shRNA knockdown of *NEK9* and *PTDSS1* in osteogenically differentiating hMSCs. We found five shRNAs that all reduced *NEK9* mRNA expression between 60% and 82% compared to the scrambled control 4 days posttransduction (Fig. 6 *A*). Four of the five shRNAs against *NEK9* inhibited mineralization by 70% or more after 3 weeks of osteogenic differentiation (Fig. 6 *B*). Although the majority of *NEK9* shRNAs had no effect on total protein levels, one shRNA did decrease total proteins levels after 3 weeks of treatment (Fig. 6 *C*). We identified five shRNAs against *PTDSS1* that all reduced mRNA expression in the range of 48% to 75% in differentiating hMSCs (Fig. 6 *D*). We observed that three out of the five shRNAs against *PTDSS1* strongly inhibited mineralization, by 67% to 92% (Fig. 6 *E*). The majority of shRNAs against *PTDSS1* had no effect on total protein levels; however, sh25‐treated hMSCs displayed increased levels of total protein after 3 weeks of culture (Fig. 6 *F*). Overall these results demonstrate that both NEK9 and PTDSS1 play a role in supporting osteoblast differentiation of hMSCs.

## Discussion

*CLIC3* was identified as a new gene specifically regulated in the osteogenic lineage of differentiating human MSCs. Lentiviral transduction‐mediated overexpression and silencing of *CLIC3* during osteogenesis revealed a crucial function for CLIC3 in promoting osteoblast mineralization. Overexpression of *CLIC3* in hMSCs strongly enhanced in vivo bone formation in a mouse model for ectopic human bone formation, further emphasizing that CLIC3 plays an important role in human osteoblast differentiation. Bioinformatics analysis of proteins identified by CLIC3‐His pull‐down suggests CLIC3s role during osteoblast differentiation may be related cytoskeletal associations and signaling, cell adhesion, and/or nuclear pore formation or transport of proteins or ions through nuclear pores. Finally, we identified that CLIC3 interacts with NEK9 and PTDSS1 during osteoblast differentiation, and inhibition of the *NEK9* and *PTDSS1* expression reduces osteogenic differentiation of hMSCs.

CLICs are a diverse group of proteins, having been associated with a wide variety of biological processes and cellular functions including: angiogenesis^33^; macrophage activation^34^; DNA damage^35^; maintenance of membrane structure^36^; bone resorption^37^; cell growth, cell division, and apoptosis^23^, ^38^, ^39^; and acidification of intracellular organelles.^33^, ^40^ All CLIC proteins contain a ∼240‐residue CLIC module that adopts a glutathione S‐transferase (GST) superfamily fold^41^; within it there is conserved cysteine residue at what looks like an enzymatic active site.^18^, ^19^, ^42^ CLIC3 was first identified based on its interaction with MAPK15, at the time known as ERK7, in a yeast two‐hybrid assay and subsequently confirmed by coimmunoprecipitation in COS cells; Qian and colleagues^23^ proposed that it plays a role in controlling mammalian cell growth based on its association with MAPK15. This is the first report of CLIC3 being involved in osteoblastic differentiation of hMSCs and human bone formation. Our in vitro results revealed the important role for CLIC3 in osteoblast differentiation and the strong induction of bone formation by hMSCs after *CLIC3* overexpression seen in our model of human ectopic bone formation in a mouse supports the strong role *CLIC3* has in ossification. It is worth noting that although this model provides an in vivo environment to study human bone formation, it is limited by the fact that the scaffold and cells are not within their native bone microenvironment and specific factors and vascularization may be lacking, resulting in lower bone formation overall. Our findings, which are in line with previous reports,^22^, ^23^ show that CLIC3 is predominately localized to the perinuclear and nuclear regions, supporting our bioinformatics findings suggesting a role for CLIC3 in the nuclear pore complex and/or export of RNA and ribonucleoprotein complexes from the nucleus. Like our results presented here, CLIC1, which shares a high degree of homology to CLIC3, with 48% to 49% of the protein sequences being identical,^(23)^ was reported to be important for murine osteoblast differentiation through MSC lineage decision‐making. CLIC1 was upregulated in response to Wnt treatment and downregulated following adipogenic treatment in murine MSCs; overexpression of *CLIC1* led to increased ALP activity and mineralization as well as increased expression of ALP, osterix, and osteocalcin.^43^ The authors showed that CLIC1 overexpression caused hyperpolarization of the mitochondrial membrane potential, suggesting that its role in osteogenic differentiation is linked to supporting energy supplementation. Based on their structural similarities, it would be possible to postulate that the two proteins may have similar functions; however, we do not have any evidence thus far that CLIC3 functions in regulating mitochondrial membrane potential or energy metabolism. Although the detailed mechanism by which CLIC3 regulates human osteoblast differentiation is still far from understood, the discovery of its interaction with NEK9 and PTDSS1 during osteogenesis does give us some clues to its function.

After discovering that NEK9 associates with CLIC3 using protein pull‐down and immunofluorescence, we confirmed that NEK9 is important in osteogenic differentiation by showing that *NEK9* knockdown leads to reduced mineralization. Very recently it was reported that recessive mutations in NEK9 cause a lethal skeletal dysplasia characterized by fetal akinesia, multiple contractures, shortened long bones, thoracic dysplasia, pulmonary hypoplasia, and protruding abdomen.^44^ This stop‐gain mutation results in a truncated protein that misses the majority of the RCC1‐like domain, all of the NEK6 interaction region, and a C‐terminal coiled‐coil domain.^44^ In fibroblasts from these patients, the *NEK9* mutation results in a number of cell‐cycle defects including reduced proliferation capability and delayed cell‐cycle progression through the G1/S boundary and S‐phase. Analysis of *NEK9* in patient fibroblasts also showed reduced cilia number and length and in *Caenorhabditis elegans* the NEK9 orthologue (NEKL‐1) expression was restricted exclusively to a subset of ciliated cells, suggesting that NEK9 is involved in ciliary function.^44^ Previous studies have shown the importance of primary cilia in osteoblast mechanosensing and function to enhance mineralization,^45^, ^46^ making this a potential mechanism through which CLIC3 interacts with NEK9; however, our CLIC3 and NEK9 immunolabelings do not appear to be specific to the primary cilia. *NEK9* is one of 11 human NIMA‐related kinase (NEK) genes that encode serine‐threonine kinases with diverse biological roles including cell‐cycle control, cilia regulation, and DNA damage sensing and repair,^47^, ^48^, ^49^, ^50^ and NEK9, specifically, is known to be involved in regulating spindle organization, chromosome alignment, cytokinesis, and normal cell‐cycle progression.^51^, ^52^ Mammalian Nek9 binds γ‐tubulin and localizes to the centrosomes and spindle poles during early cell division, functioning in the microtubule organizing center during mitosis.^50^, ^53^ Our immunofluorescence results demonstrate that both CLIC3 and NEK9 are most abundant surrounding the nucleus, suggesting that CLIC3 and NEK9 are associated with the microtubule‐organizing center. It has been reported that CLIC3 and other CLIC proteins associate with the cytoskeleton or scaffolding proteins for endosome trafficking.^20^, ^21^, ^22^ In addition, recent work in our laboratory has shown that inhibition of microtubules leading to dramatic cytoskeletal modifications and an increase in focal adhesions and BMP2 activity is sufficient to cause osteogenic differentiation of hMSCs,^28^ and osteogenic differentiation is associated with significant changes in cytoskeletal protein in human osteoblasts.^27^ In addition, our bioinformatics analysis of CLIC3‐His pulled‐down proteins identified an overrepresentation of proteins involved in processes related to cytoskeletal and integrin signaling. Collectively, we suggest that NEK9 and CLIC3 may regulate osteoblast differentiation via cytoskeleton‐associated signaling processes, but this requires further investigation.

Our results show that CLIC3 and PSS1 associate in differentiating hMSCs and that knockdown of *PTDSS1* significantly reduces osteoblast differentiation. *PTDSS1* encodes phosphatidylserine synthase 1 (PSS1) that, along with phosphatidylserine synthase 2 (PSS2), promotes the biosynthesis of phosphatidylserine (PS) by the exchange of L‐serine with the choline moiety of phosphatidylcholine.^54^ In 2014^55^ it was discovered that de novo missense mutations in the *PTDSS1* gene are responsible for Lenz‐Majewski hyperostotic dwarfism (LMHD), an extremely rare condition characterized by sclerosing bone dysplasia, intellectual disability, and distinct craniofacial, dental, cutaneous, and distal limb anomalies (OMIM #151050),^56^, ^57^ which was affirmed by Whyte and colleagues,^58^ reporting additional LMHD patients harboring mutations in *PTDSS1*. These *PTDSS1* mutations result in a gain of function phenotype leading to increased synthesis of phosphatidylserine (PS).^55^, ^58^ Bone turnover markers in a female LMHD patient, who was confirmed to have a mutation in *PTDSS1*, revealed that her osteosclerosis was a result of accelerated bone formation along with unremarkable rates of bone resorption.^58^ They also found elevated levels of phosphoserine in their LMHD patients’ urine, which could be indicative of increased PS biosynthesis. PS has unique physical and biochemical properties that lead to its physiological importance in roles related to apoptosis, coagulation, the internalization of viruses, and Ras/Rho and protein kinase C signaling.^54^, ^59^, ^60^ CLIC proteins have also been shown to be heavily involved in Rho signaling in a number of cell types,^18^, ^42^, ^53^ providing a potential functional link between the two proteins. In bone PS is known to bind calcium within matrix vesicles leading to HA crystal formation,^61^ and enhance osteogenic differentiation of MSCs^62^ and human osteoblast progenitor cells^63^ to promote bone formation. In addition, PS‐containing liposomes were shown to inhibit osteoclast differentiation and prevent trabecular bone loss,^64^ making PS a potential candidate for osteoporosis treatment. Interestingly, in cultured A2780 ovarian carcinoma cells and in pancreatic and ovarian tumors that contain elevated levels of CLIC3, CLIC3 functions to mediate the return of α5β1 from late endosomes/lysosomes to the plasma membrane.^20^ We hypothesize that CLIC3 may act similarly in osteoblasts to direct the transport of PS to the plasma membrane or to matrix vesicles. These previous findings, taken together with our current work, supports the idea that CLIC3 works together with PSS1 to increase biosynthesis of PS in osteoblast differentiation and bone formation.

In conclusion, we have successfully identified *CLIC3* to be a novel gene modulating osteoblast differentiation and enhancing bone formation. Although further studies are required to determine the molecular mechanisms by which CLIC3 modulates mineralization, we postulate that CLIC3 and NEK9 both play roles in the microtubule organizing center to induce cytoskeletal changes important for osteoblast differentiation, and that CLIC3 interacts with PSS1 to enhance PS synthesis and PS translocation to the plasma membrane where it plays an important role in matrix vesicle–mediated HA formation and mineralization of the bone. The specificity of *CLIC3* to promote the osteogenic lineage while its expression decreases during adipocyte differentiation, in combination with the importance of CLIC3 during human osteoblast differentiation, could make it a potential target for future bone anabolic treatments.

## Disclosures

All authors state that they have no conflicts of interest.

## Supporting information

Additional supporting information may be found in the online version of this article at the publisher's web‐site.

Supporting Tables S1.

Supporting Information S1.
