# Supplementary material for: Identification of Chloride Intracellular Channel Protein 3 as a Novel Gene Affecting Human Bone Formation
Source: JBMR Plus. 2017 Apr 28;1(1):16–26. doi: 10.1002/jbm4.10003 (PMC6124162; doi:10.1002/jbm4.10003)
Supplement: Supplementary file 1 — Supporting Tables S1. [file JBM4-1-16-s001.docx]

Table S1: All proteins found by CLIC3-His pull down

| Protein name | Gene ID | Ratio LFQ  CLIC3 / EV |
| --- | --- | --- |
| Chloride intracellular channel protein 3 | CLIC3 | 1227400000 |
| Serine/threonine-protein kinase Nek9 | NEK9 | 5714650 |
| 1-phosphatidylinositol 4,5-bisphosphate phosphodiesterase beta-3 | PLCB3 | 3564350 |
| Aminoacyl tRNA synthase complex-interacting multifunctional protein 1 | AIMP1 | 2891250 |
| Casein kinase II subunit alpha 3;  Casein kinase II subunit alpha | CSNK2A1;  CSNK2A3 | 2350900 |
| 40S ribosomal protein S28 | RPS28 | 1803250 |
| Phosphatidylserine synthase 1 | PTDSS1 | 1471350 |
| Nuclear pore complex protein Nup160 | NUP160 | 1106150 |
| Rho guanine nucleotide exchange factor 7 | ARHGEF7 | 2.72 |
| Lipoamide acyltransferase component of branched-chain alpha-keto acid dehydrogenase complex | DBT | 2.39 |
| Centrosomal protein of 170 kDa | CEP170 | 2.24 |
| Cytosolic acyl coenzyme A thioester hydrolase | ACOT7 | 2.20 |
| LIM domain only protein 7 | LMO7 | 2.08 |
| Splicing factor, proline- and glutamine-rich | SFPQ | 2.03 |
| cAMP-dependent protein kinase type I-alpha regulatory subunit | PRKAR1A | 2.03 |
| BTB/POZ domain-containing protein KCTD12 | KCTD12 | 2.00 |
| Serine/arginine repetitive matrix protein 1 | SRRM1 | 1.96 |
| Phostensin | PPP1R18 | 1.95 |
| Heterogeneous nuclear ribonucleoprotein D0 | HNRNPD | 1.89 |
| Actin, aortic smooth muscle;  Actin, gamma-enteric smooth muscle | ACTA2;  ACTG2 | 1.85 |
| Kinectin | KTN1 | 1.80 |
| Mesoderm-specific transcript homolog protein | MEST | 1.79 |
| Sorting nexin-33 | SNX33 | 1.78 |
| Transitional endoplasmic reticulum ATPase | VCP | 1.76 |
| Nucleoporin NUP188 | NUP188 | 1.76 |
| 5-nucleotidase | NT5E | 1.74 |
| 26S proteasome non-ATPase regulatory subunit 4 | PSMD4 | 1.72 |
| Heterogeneous nuclear ribonucleoprotein U-like protein 2 | HNRNPUL2-BSCL2 | 1.72 |
| Pinin | PNN | 1.69 |
| Heterogeneous nuclear ribonucleoprotein M | HNRNPM | 1.68 |
| Formin-binding protein 1-like | FNBP1L | 1.68 |
| RuvB-like 1 | RUVBL1 | 1.66 |
| Nexilin | NEXN | 1.66 |
| E3 SUMO-protein ligase RanBP2 | RANBP2 | 1.65 |
| LIM and SH3 domain protein 1 | LASP1 | 1.65 |
| Sorbin and SH3 domain-containing protein 2 | SORBS2 | 1.65 |
| Lamin-B2 | LMNB2 | 1.65 |
| Caveolin-1; Caveolin | CAV1 | 1.62 |
| H/ACA ribonucleoprotein complex subunit 4 | DKC1 | 1.61 |
| Importin subunit beta-1 | KPNB1 | 1.61 |
| Caldesmon | CALD1 | 1.58 |
| Ribose-phosphate pyrophosphokinase 1;  Ribose-phosphate pyrophosphokinase 2;  Ribose-phosphate pyrophosphokinase 3 | PRPS1;  PRPS2;  PRPS1L1 | 1.58 |
| Protein FAM98B | FAM98B | 1.58 |
| Plasminogen activator inhibitor 1 | SERPINE1 | 1.57 |
| THO complex subunit 2 | THOC2 | 1.55 |
| Heterogeneous nuclear ribonucleoproteins C1/C2; Heterogeneous nuclear ribonucleoprotein C-like 1; Heterogeneous nuclear ribonucleoprotein C-like 2 | HNRNPC;  HNRNPCL1;  HNRNPCL2 | 1.53 |
| Pentraxin-related protein PTX3 | PTX3 | 1.52 |
| Nuclear RNA export factor 1 | NXF1 | 1.52 |
| Alpha-parvin | PARVA | 1.52 |
| Casein kinase I isoform delta;  Casein kinase I isoform epsilon | CSNK1E;  CSNK1D | 1.51 |
| Oxysterol-binding protein 1 | OSBP | 1.51 |
| Glia-derived nexin | SERPINE2 | 1.50 |

All proteins found by His-tagged CLIC3 pull down that met our selection criteria: 1) LFQ value in CLIC3 samples of greater than 1 x 10^6^, 2) number of unique peptides covering a protein equals 3 or more, and 3) ratio of CLIC3 samples versus control samples of 1.5 or greater. Protein lysates from osteogenically differentiating hMSCs were isolated on day 5 in cultures either overexpressing His-tagged CLIC3 or transduced with empty vector (EV) control, and subjected to protein pull-down for the His-tagged CLIC3 and proteins binding to it. Proteins were determined by mass spectrometry measurements (n=2). The top proteins are listed here, ranked on their ratio of CLIC3 versus control average LFQ intensity (i.e. present in CLIC3 overexpressing condition and absent in the EV condition). LFQ: Label-free quantification.

Table S2: Canonical Pathways

| Rank | Ingenuity Canonical Pathways | p-value |
| --- | --- | --- |
| 1 | PRPP Biosynthesis I | 0.00000002 |
| 2 | Gap Junction Signaling | 0.00005623 |
| 3 | Paxillin Signaling | 0.00015136 |
| 4 | Mechanisms of Viral Exit from Host Cells | 0.00017783 |
| 5 | Integrin Signaling | 0.00019055 |
| 6 | Cellular Effects of Sildenafil (Viagra) | 0.00038019 |
| 7 | Agrin Interactions at Neuromuscular Junction | 0.00081283 |
| 8 | Caveolar-mediated Endocytosis Signaling | 0.00089125 |
| 9 | RAN Signaling | 0.00091201 |
| 10 | FAK Signaling | 0.00162181 |
| 11 | Virus Entry via Endocytic Pathways | 0.00169824 |
| 12 | Stearate Biosynthesis I (Animals) | 0.00389045 |
| 13 | RhoA Signaling | 0.00416869 |
| 14 | MSP-RON Signaling Pathway | 0.00660693 |
| 15 | Epithelial Adherens Junction Signaling | 0.00691831 |
| 16 | Amyloid Processing | 0.00812831 |

All canonical pathways determined by Ingenuity pathway analysis of the 52 proteins obtained from CLIC3-His pull down. Cutoff for selection set at p < 0.01

Table S3: Gene Ontology (GO) terms

| GO term: Biological Process | Benjamini corrected  p-value | Fold Enrichment |
| --- | --- | --- |
| RNA localization | 0.000351 | 14.16 |
| transport of virus | 0.000698 | 29.13 |
| multi-organism localization | 0.000767 | 27.55 |
| multi-organism transport | 0.000767 | 27.55 |
| ribonucleoprotein complex localization | 0.000776 | 19.03 |
| RNA export from nucleus | 0.000942 | 19.34 |
| ribonucleoprotein complex export from nucleus | 0.001107 | 20.16 |
| nuclear export | 0.002483 | 12.86 |
| establishment of RNA localization | 0.002626 | 12.93 |
| biological adhesion | 0.002811 | 3.37 |
| cell adhesion | 0.002987 | 3.38 |
| nucleic acid transport | 0.003287 | 13.14 |
| RNA transport | 0.003287 | 13.14 |
| mRNA-containing ribonucleoprotein complex export from nucleus | 0.003353 | 19.80 |
| mRNA export from nucleus | 0.003353 | 19.80 |
| endomembrane system organization | 0.003963 | 6.12 |
| viral life cycle | 0.005063 | 6.68 |
| nucleobase-containing compound transport | 0.005094 | 11.01 |
| interspecies interaction between organisms | 0.005137 | 4.03 |
| symbiosis, encompassing mutualism through parasitism | 0.005137 | 4.03 |
| multi-organism cellular localization | 0.005237 | 24.99 |
| multi-organism intracellular transport | 0.005237 | 24.99 |
| RNA processing | 0.005512 | 4.48 |
| intracellular transport of virus | 0.005597 | 25.36 |
| mRNA transport | 0.007155 | 13.59 |
| regulation of protein catabolic process | 0.008455 | 7.33 |
| positive regulation of protein catabolic process | 0.008496 | 9.26 |
| RNA splicing | 0.011931 | 6.83 |
| multi-organism cellular process | 0.014118 | 3.80 |
| mitotic cell cycle process | 0.014203 | 4.21 |
| viral process | 0.014511 | 3.83 |
| regulation of cellular component organization | 0.014704 | 2.61 |
| mitotic nuclear envelope disassembly | 0.020010 | 30.89 |
| positive regulation of catabolic process | 0.023241 | 7.12 |
| nucleocytoplasmic transport | 0.023247 | 5.80 |
| nuclear transport | 0.023529 | 5.70 |
| cell cycle process | 0.023977 | 3.25 |
| cell-cell adhesion | 0.024154 | 3.48 |
| mitotic cell cycle | 0.024168 | 3.87 |
| membrane disassembly | 0.024965 | 28.32 |
| nuclear envelope disassembly | 0.024965 | 28.32 |
| regulation of catabolic process | 0.033027 | 5.33 |
| mRNA metabolic process | 0.033389 | 4.57 |
| regulation of proteolysis | 0.047213 | 4.30 |
| GO term: Molecular Function | **Benjamini corrected**  **p-value** | **Fold Enrichment** |
| poly(A) RNA binding | 0.000003 | 4.92 |
| RNA binding | 0.000007 | 3.90 |
| cadherin binding | 0.002150 | 8.04 |
| protein binding involved in cell adhesion | 0.002475 | 8.09 |
| protein binding involved in cell-cell adhesion | 0.002786 | 8.23 |
| cadherin binding involved in cell-cell adhesion | 0.002993 | 8.51 |
| cell adhesion molecule binding | 0.022222 | 5.35 |
| organic cyclic compound binding | 0.048282 | 1.58 |
| GO term: Cellular Component | **Benjamini corrected**  **p-value** | **Fold Enrichment** |
| anchoring junction | 0.000198 | 5.47 |
| adherens junction | 0.000513 | 5.20 |
| cytosol | 0.002340 | 2.16 |
| cell junction | 0.003319 | 3.24 |
| nuclear periphery | 0.003662 | 13.61 |
| cell-cell adherens junction | 0.006602 | 6.72 |
| nuclear pore | 0.006943 | 17.22 |
| nucleoplasm | 0.022067 | 2.05 |
| nuclear body | 0.043307 | 5.47 |
| cell-cell junction | 0.044715 | 3.89 |

GO terms that were identified by DAVID bioinformatics analysis of the 52 proteins obtained from CLIC3-His pull down. Cutoff for selection set at benjamini corrected p < 0.05
