# Supplementary material for: Identification of Chloride Intracellular Channel Protein 3 as a Novel Gene Affecting Human Bone Formation
Source: JBMR Plus. 2017 Apr 28;1(1):16–26. doi: 10.1002/jbm4.10003 (PMC6124162; doi:10.1002/jbm4.10003)
Supplement: Supplementary file 2 — Supporting Information S1. [file JBM4-1-16-s002.docx]

Supporting Information

*Cell culture*

After 1 week of expansion, hMSCs were seeded into 12 well plates, in αMEM medium supplemented with 10% heat-inactivated fetal calf serum. One to two days later, to allow for attachment, osteogenic differentiation was initiated using 100 nM dexamethasone (dex) and 10 mM β-glycerophosphate. For adipogenic differentiation cells were treated with 0.1 μM dex, 60 μM indomethacin, and 0.5 mM 3-isobutyl-1-methylxanthine (IBMX). Media was refreshed every 3 or 4 days. Cell extracts were harvested at different time points during culture by scraping the cells either in phosphate-buffered saline containing 0.1% triton X-100 (PBS/Triton) and stored at -80°C for biochemical analyses or in TRIzol and stored at -20°C for gene expression analyses. Alternatively, cells seeded on poly-L-lysine-coated glass coverslips were fixed in 4% phosphate-buffered paraformaldehyde for immunocytochemical procedures.

*Lentiviral-mediated overexpression and knockdown*

To generate the *CLIC3* overexpression vector E-coli were transformed with the construct and after culture plasmid DNA isolation was performed. The CLIC3 construct was ligated into a pLenti6.3 vector, using Gateway Cloning (Life Technologies, the Netherlands) and hek cells were transfected and media containing virus was collected after 48 and 72 hours.

Lentivirus (LV) was produced by transient transfection of each LV construct into human embryonal kidney (HEK) 293T cells using a standard calcium phosphate precipitation technique. The appropriate LV vector plasmid (3 µg) and the packaging vector plasmids (9 µg of ViraPower mix (Thermo Fisher Scientific)) were cotransfected into subconfluent HEK-293T cells plated in 100-mm dishes. Viral supernatants were harvested 48 and 72 hours after transfection, filtered through a 0.45-µm pore size filter, and used immediately for hMSC transduction or stored until use at –80°C. One day prior to LV transduction, hMSC were seeded into 12-well plates. Transduction consisted of 24 hour LV incubation, after which the medium was replaced with differentiation medium and the cells were cultured until further analysis. *CLIC3* mRNA overexpression or knockdown in osteoblast differentiating hMSCs was assessed at 24 hours and 7 days (compared to empty vector or scrambled shRNA-transduced cells, respectively) and mineralization was monitored at 3 weeks after transduction. Western blotting of protein extracts collected 10 days after the start of differentiation was performed to confirm overexpression. Overexpression experiments were performed in at least duplicate in a minimum of two separate experiments in MSCs from three hMSCs donors. Knockdown experiments were performed in at least duplicate in a minimum of two separate experiments.

*Alkaline phosphatase, mineralization and protein assays*

ALP activity is determined by an enzymatic reaction, in which the ALP-mediated conversion of PNPP to PNP during 10 min at 37°C was measured. For calcium measurements, cell lysates were incubated overnight with 0.24 M HCl at 4°C. Calcium content was determined colorimetrically using a calcium assay reagent prepared by combining 1 M ethanolamine buffer (pH 10.6) with 0.35 mM 0-cresolphthalein complexone in a ratio of 1:1. ALP results were adjusted for protein content of the cell lysates. For protein measurement, 200 µl of working reagent (50 volumes BCA^TM^ reagent A and 1 volume BCA™ reagent B; Pierce, Rockford, IL) was added to 25 µl of sonicated cell lysate. The mixture was incubated for 30 min at 37°C, cooled down to room temperature (RT) and absorbance was measured at 595 nm. All measurements were performed using a Victor2 plate reader (PerkinElmer Life and Analytical Science). Staining for mineralization was performed as described previously[1]. Briefly, cells were fixed with 70% ethanol and after washing they were stained for 10-20 min with alizarin Red S solution.

*Western blotting*

Total protein was collected from 12-wells plate wells in RIPA lysis buffer (Santa Cruz), containing 10 µl 200mM PMSF/1 ml RIPA, 10 µl sodium 100mM orthovanadate/1 ml RIPA and 20 µl protease inhibitor cocktail (Santa Cruz)/1 ml RIPA. Equal amounts of protein per sample were loaded and separated by SDS-PAGE (12% SDS-polyacrylamide gels) and transferred onto a nitrocellulose membrane (Hybond-ECL, Amersham Biosciences, Buckinghamshire, U.K.). After blocking nonspecific signal with 5% bovine serum albumin (BSA) in Tris-buffered saline (TBS) with 0.1 % Tween-20, the membrane was incubated with a specific antibody against *CLIC3 (*ab56364, Abcam*)(1:500)*. Membranes were probed with the secondary antibody goat anti-mouse conjugated with Alexa Fluor 680 (1:5000, Invitrogen, Cat. A21057). Immunoreactive bands were visualized using the LI-COR Infrared Imaging System according to the manufacturer’s instructions (Odyssey Lincoln, NE).

*Immunocytochemistry*

Cells were fixed with 4% paraformaldehyde in phosphate-buffered saline (PBS) for 15 minutes at RT, washed in PBS, and excess aldehyde quenched with 10 mM ethanolamine in PBS for 5 min. Cells were then permeabilized with 0.5% Triton-X-100 in PBS for 10 minutes and blocked for 30 minutes at room temperature in PBS supplemented with 1.5% bovine serum albumin (BSA) and 0.02% Triton-X-100. Cells were incubated with primary antibody (anti-CLIC3 raised in mouse, 1:150 (ab56364, Abcam); anti-NEK9 raised in rabbit, 1:20 (11192-1-AP, Proteintech); anti-PTDSS1 raised in rabbit, 1:50 (HPA016852, Atlas Antibodies)) either overnight at 4°C or for 1 hour at RT, followed by secondary antibody (Alexa Fluor® 488 conjugated anti-mouse; Alexa Fluor® 568 conjugated anti-rabbit 1:400) for 1 hour at RT. Slides were mounted using Vectashield mounting medium containing DAPI and pictures were taken on a Zeiss Axiovert 200 MOT microscope.

*In vivo implantation assay*

5 healthy, 2-3-month-old, female, NOD.CB17-Prkdc^scid^/NCrHsd (NOD-SCID) mice (Charles River Laboratories) were used for these experiments. Animals were housed in a specific pathogen free (SPF) facility with a 12 hours day-night cycle in a controlled room with temperatures of 22±1°C and humidity of 50±5%. Mice were fed with standard rodent diet *ad libitum*. hMSCs transduced with *CLIC3* or empty vector and treated with dex for 3 days prior to being trypsinized, were loaded (5 × 10^5^) onto hydroxyl-apatite/tricalcium phosphate ceramic powder (HA-TCP, 20 mg; Zimmer, Netherlands). After overnight incubation in sterile syringes the mixture was implanted subcutaneously in the dorsal surface of NOD-SCID mice. Implants were distributed evenly between the mice and locations within the mice (behind or in front of each leg). Implants were recovered after 8 weeks and fixed in 70% ethanol. After at least 4 hours fixation, implants were dehydrated, embedded in Methyl Methacrylate (MMA), and sectioned (6 µm thick). For identification of bone formed within the pellets we performed a Goldner stain on the sections: after sections were deacrylated and rehydrated they were stained in ordered steps of Weigert Haematoxylin, Ponceau de Xylidine/Acid Fuchsine solution, Orange G/Phosphomolybdene Acid solution, 0.2% Light Green and then dehydrated before being mounted in Entellan[2]. As a result of this Goldner stain, bone matrix and fibrous tissue appears in green (bone recognizable by morphology and the bright and dense staining), osteoid in orange/red, nuclei in blue and the HA-TCP grey/brown. All quantitative measurements were performed on 2 sections of each pellet. The first was taken from the outer quarter of the pellet and the second from the core of the pellet. From each section pictures were taken with a microscope (10x magnification) covering the entire tissue and individual pictures were stitched together to recapitulate the entire cross-section. All measurements were performed in image J: ceramics areas and bone areas were determined by eye based on staining and morphology, the edges were hand drawn and the resulting pixel-measurements were calculated back to mm^2^. The observer assessing the pellets was blinded towards their identity.

*Pull-down assay*

A pull-down assay was performed to isolate proteins associating with His-tagged CLIC3 using Dynabeads (10103D, Life Technologies). Bait protein was obtained from hMSCs differentiated with dex for 5 days, following transduction with either His-tagged CLIC3 or an empty vector (EV; control). Transduced cells were washed once with DPBS after which 1X Binding/Wash Buffer (Life Technologies) containing 0.1% Triton X-100 (Sigma) was added to and the flasks were placed on a shaker for 15 min at 4°C before protein extracts were collected by scraping cells on ice. Bait protein was isolated by incubating the protein lysates with His-tagged Dynabeads for 5 minutes at 4°C before washing 4 times. Proteins interacting with His-tagged CLIC3 were obtained from non-transduced extracts of hMSCs differentiated for 5 days with dex. Differentiated hMSCs were washed once with DPBS after which 1X Pull Down Buffer (Life Technologies) was added to and the flasks and protein extracts were collected as above. These samples were added to the isolated bait protein attached to the Dynabeads for 30 min at 4°C before washing 4 times. Finally, the isolated proteins were eluted in a total of 50 µl His-elution buffer.

*Mass spectrometry*

The eluted His-tagged CLIC3 proteins and their interacting proteins, as well as the control samples, were run on NuPage Novex 4–12% Bis-Tris gel (Life Technologies). A total of 30 µl for each sample, containing 7.5 µl 4X sample buffer (Life technologies), 3 µl 10X reducing agent and 8 µg protein, diluted in 20 µl water of each samples was loaded onto the gel. Then the gels were run at 200V for one hour and washed three times with milliQ water (MQ). Proteins were stained with 50 ml Coomassie staining buffer (Bio-Rad) on a shaker for one hour and de-stained with MQ overnight. The next day, gels were fixed by incubating in 10% EtOH/ 1% Acetic Acid for 30 minutes at room temperature. Then the gels were washed three times with MQ. The 1D SDS-PAGE gel lanes were cut into 2 mm slices with an automatic gel slicer and subjected to in-gel reduction with dithiothreitol, alkylation with iodoacetamide (D4, 98%; Cambridge Isotope Laboratories Inc., Tewksbury, MA, USA), and digestion with trypsin (sequencing grade; Promega, Madison, WI, USA)[3]. Nanoflow liquid chromatography coupled to a tandem mass spectrometer (LC-MS/MS) was performed on a Series 1100 capillary LC system (Agilent Technologies, Santa Clara, CA, USA) coupled to an LTQ-Orbitrap XL mass spectrometer (Thermo Scientific, Waltham, MA, USA) operating in positive mode [4]. Peptide mixtures were trapped on a ReproSil C18 reverse-phase column (Dr. Maisch GmbH, Ammerbuch-Entringen, Germany; 1.5 cm × 100 µm, packed in house) at a flow rate of 8 µl/min. Peptide separation was performed on ReproSil C18 reversed-phase column (Dr. Maisch GmbH; 15 cm × 50 *µ*m, packed in house) using a linear gradient from 0 to 80% B [A = 0.1% formic acid; B = 80% (v/v) acetonitrile, 0.1% formic acid] in 170 min and at a constant flow rate of 200 nl/min using a splitter. The column eluent was directly sprayed into the electrospray ionization source of the mass spectrometer. Mass spectra were acquired in continuum mode, and fragmentation of the peptides was performed in a data-dependent mode.

References

1. Eijken M, Koedam M, van Driel M, Buurman CJ, Pols HAP, van Leeuwen JPTM (2006) The essential role of glucocorticoids for proper human osteoblast differentiation and matrix mineralization. Mol Cell Endocrinol 248:87–93

2. Gruber HE (1992) Adaptations of Goldner’s Masson trichrome stain for the study of undecalcified plastic embedded bone. Biotech Histochem 67:30–4

3. Wilm M, Shevchenko A, Houthaeve T, Breit S, Schweigerer L, Fotsis T, Mann M (1996) Femtomole sequencing of proteins from polyacrylamide gels by nano-electrospray mass spectrometry. Nature 379:466–9

4. Alves RDAM, Eijken M, Swagemakers S, Chiba H, Titulaer MK, Burgers PC, Luider TM, Van Leeuwen JPTM (2010) Proteomic analysis of human osteoblastic cells: relevant proteins and functional categories for differentiation. J Proteome Res 9:4688–4700
